# Type I interferons provide additive signals for murine regulatory B cell induction by *Schistosoma mansoni* eggs

**DOI:** 10.1002/eji.201847858

**Published:** 2019-05-29

**Authors:** Katja Obieglo, Alice Costain, Lauren M. Webb, Arifa Ozir‐Fazalalikhan, Shelia L. Brown, Andrew S. MacDonald, Hermelijn H. Smits

**Affiliations:** ^1^ Department of Parasitology Leiden University Medical Center Leiden The Netherlands; ^2^ Lydia Becker Institute of Immunology and Inflammation University of Manchester Manchester UK

**Keywords:** Chronic infection, Interleukin (IL)‐10, Regulatory B cells, *Schistosoma mansoni*, Type I interferons

## Abstract

The helminth *Schistosoma mansoni* (*S. mansoni*) induces a network of regulatory immune cells, including interleukin (IL)‐10‐producing regulatory B cells (Bregs). However, the signals required for the development and activation of Bregs are not well characterized. Recent reports suggest that helminths induce type I interferons (IFN‐I), and that IFN‐I drive the development of Bregs in humans. We therefore assessed the role of IFN‐I in the induction of Bregs by *S. mansoni*. Mice chronically infected with *S. mansoni* or i.v. injected with *S. mansoni* soluble egg antigen (SEA) developed a systemic IFN‐I signature. Recombinant IFN‐α enhanced IL‐10 production by Bregs stimulated with *S. mansoni* SEA in vitro, while not activating Bregs by itself. IFN‐I signaling also supported ex vivo IL‐10 production by SEA‐primed Bregs but was dispensable for activation of *S. mansoni* egg‐induced Bregs in vivo. These data indicate that although IFN‐I can serve as a coactivator for Breg IL‐10 production, they are unlikely to participate in the development of Bregs in response to *S. mansoni* eggs.

## Introduction

The helminth *Schistosoma mansoni* induces a network of regulatory immune cells during the chronic phase of infection 1. The induction of B cells with regulatory properties, so called regulatory B cells (Bregs), by *S. mansoni* has been studied extensively 2, 3, 4, 5. Bregs as part of the regulatory network play an important role in limiting immunopathology and attenuate responses to bystander antigens such as allergens 6. Breg induction, as observed during chronic infection, can be replicated by in vitro stimulations with soluble egg antigens (SEAs) 7, 8 and the even single, egg‐derived molecule IPSE/alpha‐1 8 in the absence of infection. Although it is currently unclear which receptors and pathways *S. mansoni*‐derived molecules engage, factors consistently reported to be important for Breg development and activation are stimulation through the BCR 9, 10, 11, 12, CD40 9, 13, 14, 15, 16, and the toll‐like receptors (TLR) TLR2/4 17, 18, 19, TLR7 20, and TLR9 17. Moreover, different cytokines including IL‐21 21, IL‐35 22, 23, BAFF 24, 25, APRIL 26, and type I interferons (IFN‐I) 27 have been described to support Breg development.

IFN‐I are a large family of cytokines, containing 14 IFN‐α subtypes and a single IFN‐β, central in the immune response to viral infections 28. Induced, among others, by ligation of PRRs of immune and nonimmune cells, IFN‐I act in an auto‐ and paracrine manner to induce an antiviral state, but can also interfere with innate and adaptive immune responses 29, 30. IFN‐I can enhance antigen presentation and chemokine production in innate cells, promote effector T‐cell responses and induce B‐cell antibody production in viral infection (reviewed in 30). The role of IFN‐I in bacterial, fungal, and intracellular parasitic (mainly *Leishmania*, *Plasmodium*, and *Trypanosoma* spp.) infections is complex, with possible beneficial and detrimental outcomes for the host (reviewed in 28). Only recently, reports have highlighted the potential of helminths or their products to induce IFN‐I in mouse models. Infection with the gastrointestinal helminth *Heligmosomoides polygyrus* has been shown to induce IFN‐I signaling in gut and lung in a microbiota‐dependent manner, protecting mice from RSV infection 31. *Schistosoma mansoni* eggs and SEAs have been shown to induce an IFN‐I signature both in splenic DCs and in in vitro differentiated bone marrow DCs (BMDCs) 32, 33, and *Nippostrongylus brasiliensis* induces IFN‐I in skin DCs 34. A more generalized expression of IFN‐stimulated genes (ISGs) in response to *S. mansoni* products has so far only been shown by Webb et al. for whole lung tissue following i.p. sensitization and i.v. challenge with *S. mansoni* eggs 33.

B cells express the IFN‐α/‐β receptor (IFNAR) and respond to IFN‐I 35, 36, 37. B‐cell responses to IFN‐I are most extensively studied in autoimmunity. In systemic lupus erythematosus (SLE), IFN‐I are considered to promote the activation of autoreactive B cells, maturation into plasmablasts, and autoantibody production, contributing to disease pathology 38. Menon et al. add important knowledge to the picture by showing that plasmacytoid DCs (pDCs) drive the formation of IL‐10‐producing Bregs by IFN‐α production and CD40 ligation in healthy individuals, but fail to do so in SLE patients. Although Breg‐derived IL‐10 normally provides an important feedback loop that limits IFN‐α production, SLE patients have hyperactivated pDCs that fail to induce Bregs, possibly due to Bregs being less responsive to supraoptimal concentrations of IFN‐α 39. In patients with certain types of MS, IFN‐β therapy is a treatment option commonly applied. It has been reported that IFN‐β therapy not only increased IL‐10 production by monocytes and T cells 40, 41, but also B cells and plasmablasts 42.

Although Bregs can be induced by *S. mansoni*‐derived antigens in vitro, this is less potent than the induction of Bregs during chronic infection, and the induction of Bregs by IPSE/alpha‐1 has only been demonstrated in vitro 8. Helminth infections trigger a multitude of different immune responses in the host in vivo, and it is likely that additional signals, in addition to helminth molecules, are required for optimal Breg induction. Here, we sought to address whether IFN‐I are central to the induction of Bregs by *S. mansoni*. We show that both *S. mansoni* infections and i.v. injections with SEA induce a systemic IFN‐I signature in vivo. Recombinant IFN‐α enhanced B‐cell IL‐10 production in response to SEA and SEA+aCD40 in vitro, while blocking antibodies against IFNAR alpha chain (IFNAR1) reduced the ex vivo IL‐10 production by in vivo‐primed B cells. However, B‐cell induction in response to egg administration in vivo was not affected in IFNAR^−/−^ mice. Collectively, these data show that IFN‐I provide additive signals for Breg induction by *S. mansoni* in vitro, but are not crucial for *S. mansoni*‐induced Bregs in vivo.

## Results

### *Schistosoma mansoni* infections and SEA injections induce a systemic IFN signature in vivo

**Figure 1:**
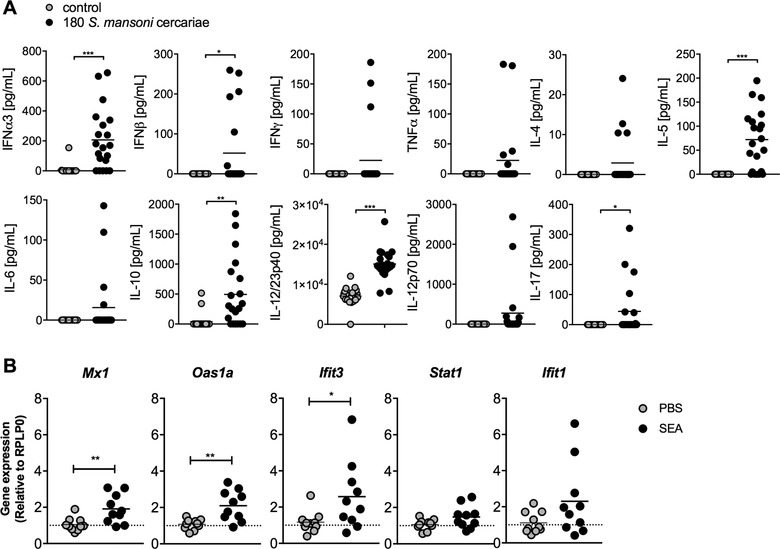
*Schistosoma mansoni* infections and SEA injections induce a systemic type I IFN signature. (A) Mice were infected with 180 *S. mansoni* cercariae and serum samples taken at day 49 of infection for assessment of cytokine levels by ELISA/CBA. Pooled data from two experiments, *n* = 20/group. (B) Splenocytes from SEA‐injected mice were harvested 12 h postinjection. The mRNA expression of ISGs was evaluated by quantitative PCR (normalized against RPLP0). Data shown are from two experiments and presented as mean +SEM, *n* = 2–8 per group. Significant differences were determined by unpaired *t*‐test. *p < 0.05, **p < 0.01, ***p < 0.001.

We first sought to assess whether chronic *S. mansoni* infection induces a systemic IFN‐I signature. High‐dose infection with 180 *S. mansoni* cercariae significantly increased the serum concentration of IFN‐α3 in the majority of animals (Fig. 1A), whereas lower doses of 20–80 cercariae did not (Fig. S1). Systemic levels of IL‐5 and IL‐12/23p40 were similarly increased, whereas IFN‐β, IL‐10, and IL‐17 were only elevated in a minority of animals (Fig. 1A). The production of IFN‐I subtypes is often difficult to assess, as they are frequently produced at low levels and transiently, or consumed by neighboring cells following production, which might explain the high dose of infection necessary to reliably detect IFN‐I in the serum. Irrespective, the significant increase in serum IFN‐I following high‐dose infection supports the notion that *S. mansoni* induces a systemic IFN‐I signature.

**Figure 2:**
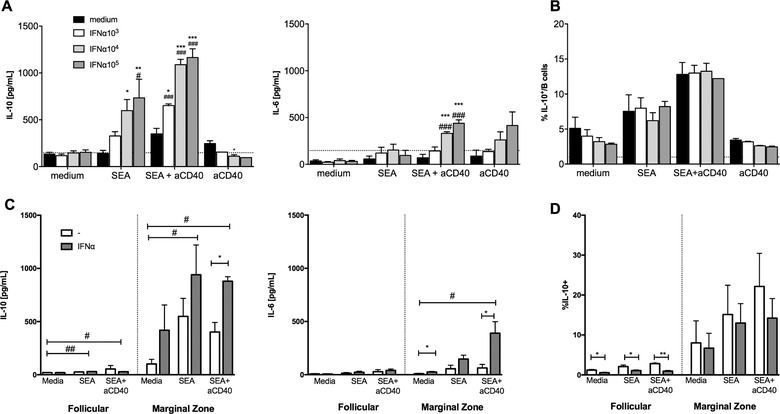
Recombinant IFN‐α enhances SEA/aCD40‐induced B‐cell IL‐10 production. (A–B) B cells were isolated from the spleen of naϊve mice and stimulated in vitro with SEA (20 µg/mL), anti‐CD40 (0.5 µg/mL), and IFN‐α (10^3^–10^5^_ _U/mL) as indicated. After 3 days of culture, supernatants were analyzed for IL‐10 and IL‐6 concentration by ELISA **(A)**, and the percentage of IL‐10^+^ B cells assessed by flow cytometry (B). (C–D) Splenic marginal zone and follicular B cells from naïve mice were sorted using flow cytometry and cultured for 2 days in the presence of SEA (20 µg/mL), anti‐CD40 (0.5 µg/mL), and IFN‐α (10^4^/mL). IL‐6 and IL‐10 production as measured by CBA (C) and frequency of IL‐10^+^ cells in each respective subset as determined by flow cytometry (D). Data are pooled from four (A) or three (B–D) experiments; each data point is the mean of two to four technical replicates. Data are presented as mean +SEM. Significant differences are indicated by *p < 0.05, **p < 0.01, ***p < 0.001 and determined by one‐way ANOVA followed by Dunnett's multiple comparisons test. ^#^ *p* < 0.05, ^##^ *p* < 0.01, ^###^ *p* < 0.001 indicate significant difference relative to medium only control.

Next, to investigate the contribution of egg products to IFN‐I induction, we examined the expression of ISGs in the splenocytes of mice i.v. injected with SEA after 12 h (Fig. 1B). SEA‐treated mice demonstrated a clear IFN‐I signature, with significant upregulation shown for the *Mx1* and *Oas1a*, and a trend toward enhanced expression for *Ifit3* and *Ifit1*. Additionally, SEA treatment induced *Oas1a* expression to a similar level to that observed for IFN‐α‐treated control mice (Fig. 2). Collectively, these data suggest that egg exposure alone, in the absence of worms, is sufficient to drive IFN‐I responses.

### Recombinant IFN‐α enhances SEA/aCD40‐induced B‐cell IL‐10 production in vitro

We have previously demonstrated that SEA induces B‐cell IL‐10 production and that CD40 ligation enhances SEA‐induced Breg development 8, whereas others have reported a synergistic effect of IFN‐α and CD40 ligation on the development of IL‐10‐producing human B cells 39. We therefore tested the effect of simultaneous stimulation of splenic B cells with SEA, anti‐CD40, and recombinant IFN‐α in vitro. After 3 days of culture, the concentration of IL‐10 in culture supernatants of SEA‐stimulated B cells increased with increasing doses of IFN‐α, whereas IFN‐α alone had no effect (Fig. 2A; Fig. S3B for gating scheme). The strongest induction of B‐cell IL‐10 production could be observed when cells were co‐stimulated with SEA and anti‐CD40 compared to SEA alone (Fig. 2A). IFN‐α at concentrations of 10^3^–10^4^ U/mL (equivalent to circa 15–150 ng/mL) significantly enhanced IL‐10 production in response to SEA and SEA+anti‐CD40, whereas IL‐10 production seemed to plateau at 10^5^ U/mL IFN‐α (Fig. 2A). IL‐10 production after co‐stimulation with IFN‐α increased up to fourfold compared to the control condition without addition of IFN‐α. IFN‐α also enhanced IL‐6 production, a proinflammatory cytokine known to be produced by B cells, in response to SEA and anti‐CD40, albeit to a lesser extent (Fig. 2A). This indicated a pattern of cytokine expression characteristic for Bregs. Conversely, the percentage of IL‐10‐producing B cells after 3 days of stimulation with SEA or SEA+anti‐CD40 in the presence of IFN‐α did not increase (Fig. 2B), which suggests that the peak of the stimulatory activity of IFN‐α occurs early and has passed, possibly due to a decline in the IFN‐α concentration in culture supernatant due to consumption, when the intracellular staining was performed after 3 days of culture. As a control, we also stimulated B cells with CpG ODN1826 (class B) and IFN‐α. Already a low concentration of 10^3^ U/mL IFN‐α strongly amplified the CpG ODN1826‐induced cytokine production (Fig. S4A) and percentage of IL‐10‐producing B cells (Fig. S4B). These data show that IFN‐α provides additional signals for the induction of B‐cell IL‐10 production in cells activated with known Breg‐inducing stimuli SEA or CpG ODN1826.

To identify which B‐cell subset produces IL‐10 in response to IFN‐α stimulation, splenic B cells were sorted into the two main subsets, CD23^low^CD21^+^ marginal zone (MZ) B cells and CD23^hi^CD21^−^ follicular (FO) B cells, for subsequent 2‐day in vitro stimulation with SEA, anti‐CD40, and recombinant IFN‐α (10^4^ U/mL; Fig. S3B for gating scheme). Unlike their FO counterparts, MZ B cells reacted potently to the addition of IFN‐α to the culture media (Fig. 2C), with IFN‐α‐treated MZ B cells demonstrating a fourfold increase in IL‐10 secretion compared to MZ B cells cultured in media alone. Importantly, the effect of IFN‐α stimulation was further potentiated by the addition of SEA or SEA+antiCD40 to the culture media, with the highest production of IL‐10 shown for MZ B cells cultured in the presence of IFN‐α, SEA, and antiCD40. In contrast, FO B cells produced relatively little IL‐10 irrespective of IFN‐α, SEA, or SEA+antiCD40 stimulation. In comparison to unstimulated FO B cells, FO B cells treated with IFN‐α and SEA ± antiCD40 produced significantly higher amounts of IL‐10. However, these IL‐10 levels were considerably lower to that produced by MZ B cells under the same stimulation conditions. Like that observed for total B cells (Fig. 2B), the percentage of IL‐10‐producing MZ B cells remained unaltered by IFN‐α stimulation (Fig. 2D), supporting the notion that peak time of IFN‐α‐stimulatory activity has already been reached. On the other hand, the percentage of IL‐10‐producing FO B cells even appeared to decrease following IFN‐α stimulation (Fig. 2D). As for IL‐6, only MZ B cells but not FO B cells showed IL‐6 production following IFN‐α stimulation (Fig. 2C). However, similar to that described for total B cells (Fig. 2A), these IL‐6 levels were considerably lower than that detected for IL‐10. Altogether, these data demonstrate a heightened responsiveness of MZ B cells to IFN‐α and schistosome antigen stimulation, and support the notion that IFN‐I and schistosome antigens synchronously drive Breg activity in vitro.

### IFNAR1 signaling provides co‐signals for IL‐10 production by in vivo‐primed B cells

**Figure 3:**
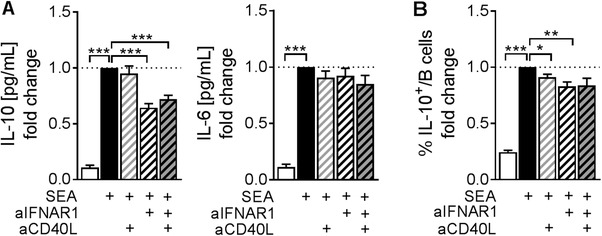
Ex vivo block of IFNAR1 reduces B‐cell IL‐10 production. Splenocytes from SEA‐injected mice (100 µg SEA i.p. on day 0 and day 7; cull day 14) were restimulated ex vivo with SEA (20 µg/mL) for 2 days in the presence or absence of anti‐CD40L (10 µg/mL) and anti‐IFNAR1 (10 µg/mL) blocking antibodies as indicated. After 2 days of culture, supernatants were analyzed for IL‐10 and IL‐6 concentration by ELISA (**A**), and the percentage of IL‐10^+^ B cells assessed by flow cytometry (**B**). Data are pooled from two experiments, *n* = 10/group. Data are presented as mean + SEM. Significant differences were determined by RM‐one way ANOVA & Dunnett's posttest comparing all groups to the SEA‐stimulated positive control. *p < 0.05; **p < 0.01; ***p < 0.001.

To assess whether IFN‐I signaling provides important signals for IL‐10 production by in vivo‐primed Bregs, we treated mice with SEA i.p. and subsequently restimulated total splenocyte cultures ex vivo with SEA, in the presence or absence of blocking antibodies against IFNAR1. We also used blocking antibodies against CD40 ligand (CD40L) upon ex vivo restimulation to assess the importance of CD40 co‐ligation on B cells for IL‐10 induction. Although blocking CD40L alone, or in combination with blocking IFNAR1, had either no or no additional effect, blocking IFNAR1 signaling significantly reduced the concentration of IL‐10 in 2‐day culture supernatants (Fig. 3A). The production of IL‐6 was not affected by either of the blocking agents (Fig. 3A), whereas the percentage of IL‐10 producing B cells in culture was mildly but significantly reduced by both blocking agents (Fig. 3B). We concluded that signaling via IFNAR1, but not the ligation of CD40, is essential for SEA‐induced B‐cell IL‐10 production in this setting.

### IFNAR1 signaling is dispensable for Breg induction in vivo

**Figure 4:**
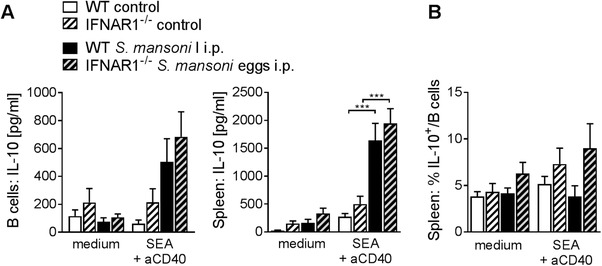
IFNAR1 signaling is dispensable for Breg induction in vivo. Splenocytes and MACS‐isolated CD19^+^ B cells from *S. mansoni* egg‐injected mice (5000 *S. mansoni* eggs i.p. on day 0 and day 7; section day 14) were restimulated ex vivo with SEA (20 µg/mL) and anti‐CD40 (2 µg/mL) for 2 days. After 2 days of culture, supernatants of isolated B cell and total splenocyte cultures were analyzed for IL‐10 concentration by ELISA (A), and the percentage of IL‐10^+^ B cells within splenocyte cultures assessed by flow cytometry (B). Data are pooled from two experiments, *n* = 8–10/group. Data are presented as mean + SEM. Significant differences were determined by one‐way ANOVA followed by Tukey's multiple comparisons test. **p < 0.01; ***p < 0.001.

To assess whether IFN‐I signaling provides important signals for Breg development and IL‐10 production in response to *S. mansoni* egg products not only in vitro but also in vivo, we induced Breg development by two doses of i.p. administered *S. mansoni* eggs (5000) in WT control or IFNAR1^−/−^ mice, a model we previously showed to be very suitable to demonstrate schistosome‐induced splenic Breg development 8. The absence of IFNAR1 did not affect the concentration of IL‐10 in B cells and total splenocyte culture supernatants in response to restimulation with SEA and anti‐CD40 (Fig. 4A). In addition, the percentage of IL‐10^+^ B cells seemed increased rather than decreased in IFNAR1^−/−^ mice (Fig. 4B). Additionally, no changes in IL‐10 production could be observed when blocking IFNAR1 signaling by means of in vivo administration of anti‐mouse IFNAR1‐blocking antibody (Fig. S5). Thus IFNAR1 signaling seems to be dispensable for the induction of Bregs to *S. mansoni* egg challenge in vivo.

## Discussion

In this study, we sought to address whether IFN‐I might provide the “missing link,” synergizing with *S. mansoni‐*derived signals for the induction of Breg IL‐10 production. We show that although *S. mansoni* infections and injections with egg antigens induce systemic IFN‐I signature, and IFN‐I signaling enhances in vitro IL‐10 production by Bregs exposed to *S. mansoni* antigens, IFN‐I responsiveness is ultimately dispensable for Breg induction by *S. mansoni* eggs in vivo.

We and others have previously shown that chronic *S. mansoni* infection induces Bregs 3, 4, 43, 44, and that this Breg‐inducing effect can be replicated by isolated eggs, SEA, and even the single, egg‐derived molecule IPSE/alpha‐1 in the absence of adult worms and a natural infection 7, 8. Components of SEA directly bind to splenic B cells 8, but the receptors ligated and signaling pathways activated by these antigens remain to be identified. Moreover, SEA immunization is less potent than chronic infection at Breg induction in vivo, and the induction of Bregs by IPSE/alpha‐1 has only been demonstrated in vitro 8. Helminth infections trigger a multitude of different immune responses in the host in vivo, and it is likely that additional signals, in addition to helminth molecules, are required for optimal Breg induction.

We found an increased concentration of IFN‐I in serum of mice actively infected with *S. mansoni*, and enhanced expression of ISGs in the spleen of SEA‐injected mice. These data are in line with previous reports on the capacity of *S. mansoni* eggs or egg antigens, *H. polygyrus* infection, and *N. brasiliensis* antigens to induce IFN‐I 31, 32, 33, 34. pDCs are considered an important source of IFN‐I 45. IFN‐I were, however, produced by conventional DCs (cDCs) rather than pDCs after SEA stimulation of BMDCs in vitro 33. We have not addressed the cellular source of IFN‐I in our study; therefore, both pDCs and cDCs remain possible sources. Notably, little is reported to date regarding IFN‐I production by human DCs in response to helminths, but work of our own group suggests that *S. mansoni* egg antigens do not induce IFN‐I in human monocyte‐derived DCs (Everts, personal communication).

We show here that recombinant IFN‐α, while having no measurable effect on its own, significantly and dose‐dependently increased IL‐10 production by B cells in response to in vitro stimulation with SEA alone or SEA+aCD40. Additionally, in harmony with previous mechanistic studies 8 and models of chronic *S. mansoni* infection 46, we provide evidence that MZ B cells, as opposed to FO B cells, are responsible for this increase in IL‐10. IFN‐α was also shown to have a synergistic effect on SEA+aCD40‐induced IL‐6 production, albeit to lesser extent. Conversely, the percentage of IL‐10^+^ B cells was unchanged or slightly reduced after 3 days of culture in the presence of increasing amounts of IFN‐α, suggesting that IFN‐I may change the dynamics and timing of IL‐10 production. Menon et al. observe an optimal IL‐10 induction in naïve TLR9‐stimulated B cells at 50 × 10^5^ U/mL IFN‐α and a less effective stimulation at higher concentrations 39, whereas we find an additive effect even at 1 × 10^6^ U/mL on both SEA‐ and TLR9‐stimulated B cells on IL‐10 concentration in culture supernatants. The fact that IFN‐α has no effect at all on IL‐10 or IL‐6 expression by itself underpins that IFN‐I signaling modulates responses in preactivated B cells rather than providing an activation signal to B cells by itself, which has been similarly reported by others 27, 42. In this context, it is plausible that stimulation with *S. mansoni*‐derived antigens in vitro provides this preactivation signal, rather than SEA‐ and IFN‐I‐specific signaling pathways synergizing to promote B‐cell IL‐10 production. This is in line with previous reports describing IFN‐I signaling to regulate B‐cell responses to other preactivating stimuli such as BCR or TLR7 ligation 35, 36. In this context, Braun et al. show that murine, mature splenic B cells get partially activated by treatment with IFN‐α/‐β, characterized by the upregulation of activation markers and increased survival in the absence of proliferation or terminal differentiation, and display enhanced response to BCR ligation 35. Poovassery and Bishop report that both BCR and IFNAR signaling restore TLR7‐induced B‐cell hyporesponsiveness 36. The percentage IL‐10^+^ B cells that tends to decrease at the end of culture might suggest that the peak of IFN‐α stimulatory activity has occurred earlier and that after 3 days of culture the IFN‐I concentration in culture supernatant has already declined, making an earlier time point for the assessment of IL‐10^+^ B cells preferable.

Arguably, in vitro stimulation of isolated B cells with recombinant IFN‐α does not mimic the natural situation very well. We therefore also assessed the role of IFN‐I signaling on Breg recall responses ex vivo. Blocking IFNAR1 upon ex vivo restimulation of in vivo SEA‐induced Bregs significantly reduced IL‐10, but not IL‐6 production. Adding blocking antibody against CD40L to the cultures, and thereby preventing the ligation of CD40 expressed on B cells by accessory cells present in whole splenocyte cultures, had only negligible effects. This might indicate that although CD40 ligation has previously been shown to enhance B‐cell IL‐10 expression 8, 9, 15, it does not provide additional signals for B‐cell IL‐10 production in this restimulation setting. This might point at a difference in the contribution of CD40 signaling to Breg induction upon concurrent priming of B cells with an antigen and agonistic anti‐CD40 8 and upon ex vivo restimulation as performed in this study. Alternatively, it is also possible that insufficient CD40 ligation was occurring in our chosen culture conditions, and so explains why blocking CD40 had a negligible effect. Finally, although injections with *S. mansoni* egg products can effectively drive both systemic IFN‐I responses and Breg development 8, we found B‐cell IL‐10 production to be unaltered in IFNAR1^−/−^ mice upon egg i.p. administration, suggesting that IFN‐I signaling is dispensable in this setting. However, it is unknown whether other signals have compensated for the lack of IFN‐I, and so, a full blown Breg response is still driven in IFNAR^−/−^ mice. In addition, we have not tested the role of IFN‐I during schistosome infections in IFNAR^−/−^ mice and therefore cannot exclude that IFN‐I produced in response to repetitive stimulations and chronic inflammation may still contribute to Breg development during schistosomiasis. Combined, our data suggests that in vivo, where multiple pathways are activated simultaneously and potentially act synergistically, IFN‐I signaling is not essential for the development and activation of Bregs against *S. mansoni* eggs.

The physiological role of IFN‐I in helminth infections has not been extensively studied to date. Enteric *H. polygyrus*‐induced IFN‐I protects from RSV coinfection 31. SEA‐stimulated BMDCs induce IFN‐I 32, and SEA‐stimulated cDCs as well as skin DCs exposed to *N. brasiliensis* were shown to be dependent on IFN‐I signaling for their effective induction of Th2 response 33, 34. Therefore, more research is needed to fully understand the role of IFN‐I in helminth, and more specifically in *S. mansoni* infections.

Collectively, the data presented here show that although IFN‐I can enhance IL‐10 production by *S. mansoni*‐activated Bregs both in vitro and ex vivo, IFN‐I signaling is dispensable for the formation and activation of *S. mansoni*‐induced Bregs in vivo. A better understanding of the signals for optimal Breg development and activation is required to develop novel therapies around Bregs.

## Material and methods

### Animals

Female C57BL/6 mice (Harlan) were housed under SPF conditions in the animal facility of the Leiden University Medical Center (Leiden, The Netherlands). *Ifnar1^−/−^* mice on a C56BL/6 background were housed at the University of Manchester. All animals were used for experiments at 6–12 weeks of age. All animal studies were performed in accordance with either the Animal Experiments Ethical Committee of the Leiden University Medical Centre or under a license granted by the home office (UK) in accordance with local guidelines.

### Preparation of SEA and eggs

*S. mansoni* eggs were isolated from trypsinized livers or guts of hamsters after 50 days of infection, washed in RPMI medium supplemented with penicillin (300 U/mL), streptomycin (300 µg/mL), and amphotericin B (300 µg/mL) and stored at –80°C until use. SEA was prepared as previously described 47. Protein concentration was determined by BCA. SEA preparations were routinely tested for endotoxin contamination by Limulus Amoebocyte Lysate assay or TLR4‐transfected HEK reporter cell lines.

### *S. mansoni* infections and in vivo injections

For the high‐dose infection model, mice were percutaneously infected with approximately 180 cercariae and serum collected on day 49 after infection. For the evaluation of splenic ISG expression in SEA‐/IFN‐α‐treated mice, mice were i.v. injected with 50 µg SEA in PBS or intraperitoneally injected with PBS or IFN‐α (20 × 10^3^ units). Splenocytes were harvested 12 h after injection, snap‐frozen, and stored –80°C for later analysis. For the egg challenge model in IFNAR^−/−^ mice, mice received two intraperitoneal injections (day 0 and day 7) of 5000 *S. mansoni* eggs diluted in sterile PBS. Mice were sacrificed 7 days after the last injection.

### Splenocyte and B‐cell isolation

Spleens were homogenized by passage through a 70 µM cell strainer (BD Biosciences) and erythrocytes depleted from the single cell suspension by lysis. B cells were purified from splenocytes by anti‐CD19 MicroBeads (Miltenyi Biotech) following the manufacturer's instructions. For cell sorting experiments, MACs‐isolated CD19^+^ B cells were sorted by flow cytometry into FO B cells (CD23^+^CD21^low^) and MZ B cells (CD23^−^CD21^hi^).

### In vitro stimulation

Splenic CD19^+^ B cells, MZ B cells, and FO B cells (1.5 × 10^6^/mL) were cultured in medium (RPMI 1640 GlutaMAX; Thermo Fisher Scientific) supplemented with 5% heat‐inactivated FCS (Greiner Bio‐One), 2‐mercaptoethanol (5 × 10^−5^ M), penicillin (100 U/mL), and streptomycin (100 µg/mL; all Sigma‐Aldrich). Cells were stimulated with the following stimuli as indicated in the figures: SEA (20 µg/mL), aCD40 (clone 1C10; 0.5 µg/mL; Biolegend), recombinant IFN‐α (Biolegend), CpG ODN 1826 (class B; 0.2–1 µM; Invivogen), aCD40L‐blocking antibody (clone MR1; 10 µg/mL; kind gift from L. Boon, Bioceros), and aIFNAR1‐blocking antibody (clone MAR1‐5A3; 10 µg/mL; eBioscience). After 2 days (FO and MZ B cells) or 3 days (total B cells) culture at 37°C, supernatants were harvested for cytokine analysis by ELISA or cytometric bead array (CBA). For flow cytometric analysis of IL‐10, cells were restimulated with PMA (100 ng/mL) and ionomycin (1 µg/mL) for 4 h in the presence of Brefeldin A (10 µg/mL; all Sigma–Aldrich).

### Flow cytometry

Cells were stained with antibodies against B220 (clone RA3‐6B2), CD21 (clone 7G6), CD23 (clone B3B4), and IL‐10 (clone JESS‐16E3). Dead cells were stained with live/dead fixable aqua dead cell stain kit (Thermo Scientific). FcγR‐binding inhibitor (2.4G2, kind gift of L. Boon, Bioceros) was added to all stainings. Flow cytometry was performed on a FACS Canto II using FACSDiva software (BD Biosciences) followed by data analysis using FlowJo.

### ELISA and CBA

The concentration of IL‐6 and IL‐10 in cell‐free culture supernatants was assessed by OptEIA ELISA kits (BD Biosciences) (total B cells) or BD cytometric bead array Flex‐set kits (BD Biosciences; MZ and FO B cells). The concentration of cytokines in serum of chronically infected mice was also assessed by CBA Flex‐set kits, except for IFN‐α3 and IFN‐β that were measured by ELISA (PBL).

### RNA extraction and quantitative PCR analysis

RNA from frozen splenocytes was extracted using TriPure isolation reagent (Roche) and translated to cDNA using SuperScript™ III Reverse Transcriptase and Oligo (dT; Life Technologies). Quantitative PCR was performed using SYBR Green Master Mix (Applied Biosystems) using a Biorad CFX96 Real‐time system C1000 thermal cycle. Expression levels were normalized to *Gadph*. The following primers were used:

*RPLP0*: 5′‐ TCTGGAGGGTGTCCGCAACG–3′ 5′‐ GCCAGGACGCGCTTGTACCC‐3′; *MX1*: 5′‐TTCAAGGATCACTCATACTTCAGC–3′ 5′‐GGGAGGTGAGCTCCTCAGT‐3′; *Oas1a*: 5′‐GCTGCCAGCCTTTGATGT–3′ 5′‐TGGCATAGATTGTGGGATCA‐3′;
*Ifit3* 5′‐TGAACTGCTCAGCCCACA–3′ 5′‐TCCCGGTTGACCTCACTC‐3′;
*Stat11* 5′‐GTGCCTCTGGAATGATGGGT–3′ 5′‐GAAGTCAGGTTCACCTCCGT‐3′;
*Ifi30* 5′‐GAACATGGTGGAGGCCTGTC–3′ 5′‐TGGCGCACTCCATGATACTC‐3′;
*Ifit1* 5′‐TCTAAACAGGGCCTTGCAG–3′ 5′‐GCAGAGCCCTTTTTGATAATGT‐3′

### Statistical analysis

Statistical analysis was performed using GraphPad Prism (version 7.02). All data are presented as mean ± SEM. *p*‐Values < 0.05 were considered statistically significant.

## Conflict of interest

The authors declare no financial or commercial conflict of interest.

**Abbreviations**
Breg: regulatory B cell
cDCs: conventional DCs
FO: follicular
IFN‐I: type I interferon
IFNAR: IFN‐α/‐β receptor
MZ: marginal zone
pDCs: plasmacytoid DCs
SEA: soluble egg antigen
SLE: systemic lupus erythematosus

## Supporting information

Supplemental Information

## References

[1] McSorley, H. J. and Maizels, R. M. , Helminth infections and host immune regulation. Clin. Microbiol. Rev. 2012 25: 585–608. 23034321 10.1128/CMR.05040-11 PMC3485755

[2] Jankovic, D. , Cheever, A. W. , Kullberg, M. C. , Wynn, T. A. , Yap, G. , Caspar, P. , Lewis, F. A. et al., CD4+ T cell‐mediated granulomatous pathology in schistosomiasis is downregulated by a B cell‐dependent mechanism requiring Fc receptor signaling. J. Exp. Med. 1998 187: 619–629. 9463412 10.1084/jem.187.4.619 PMC2212140

[3] Amu, S. , Saunders, S. P. , Kronenberg, M. , Mangan, N. E. , Atzberger, A. and Fallon, P. G. , Regulatory B cells prevent and reverse allergic airway inflammation via FoxP3‐positive T regulatory cells in a murine model. J. Allergy Clin. Immunol. 2010 125: 1114–1124.e8. 20304473 10.1016/j.jaci.2010.01.018

[4] van der Vlugt, L. E. , Labuda, L. A. , Ozir‐Fazalalikhan, A. , Lievers, E. , Gloudemans, A. K. , Liu, K. Y. , Barr, T. A. et al., Schistosomes induce regulatory features in human and mouse CD1d(hi) B cells: inhibition of allergic inflammation by IL‐10 and regulatory T cells. PLoS One 2012 7: e30883. 22347409 10.1371/journal.pone.0030883 PMC3275567

[5] Finlay, C. M. , Walsh, K. P. and Mills, K. H. , Induction of regulatory cells by helminth parasites: exploitation for the treatment of inflammatory diseases. Immunol. Rev. 2014 259: 206–230. 24712468 10.1111/imr.12164

[6] Maizels, R. M. and Yazdanbakhsh, M. , Immune regulation by helminth parasites: cellular and molecular mechanisms. Nat. Rev. Immunol. 2003 3: 733–744. 12949497 10.1038/nri1183

[7] Tian, F. , Hu, X. , Xian, K. , Zong, D. , Liu, H. , Wei, H. , Yang, W. et al., B10 cells induced by Schistosoma japonicum soluble egg antigens modulated regulatory T cells and cytokine production of T cells. Parasitol. Res. 2015 114: 3827–3834. 26149531 10.1007/s00436-015-4613-x

[8] Haeberlein, S. , Obieglo, K. , Ozir‐Fazalalikhan, A. , Chaye, M. A. M. , Veninga, H. , van der Vlugt, L. , Voskamp, A. et al., Schistosome egg antigens, including the glycoprotein IPSE/alpha‐1, trigger the development of regulatory B cells. PLoS Pathog. 2017 13: e1006539. 28753651 10.1371/journal.ppat.1006539 PMC5550006

[9] Fillatreau, S. , Sweenie, C. H. , McGeachy, M. J. , Gray, D. and Anderton, S. M. , B cells regulate autoimmunity by provision of IL‐10. Nat. Immunol. 2002 3: 944–950. 12244307 10.1038/ni833

[10] Miles, K. , Heaney, J. , Sibinska, Z. , Salter, D. , Savill, J. , Gray, D. and Gray, M. , A tolerogenic role for toll‐like receptor 9 is revealed by B‐cell interaction with DNA complexes expressed on apoptotic cells. Proc. Natl. Acad. Sci. USA 2012 109: 887–892. 22207622 10.1073/pnas.1109173109 PMC3271931

[11] Yanaba, K. , Bouaziz, J. D. , Matsushita, T. , Tsubata, T. and Tedder, T. F. , The development and function of regulatory B cells expressing IL‐10 (B10 cells) requires antigen receptor diversity and TLR signals. J. Immunol. 2009 182: 7459–7472. 19494269 10.4049/jimmunol.0900270 PMC3733128

[12] Matsumoto, M. , Fujii, Y. , Baba, A. , Hikida, M. , Kurosaki, T. and Baba, Y. , The calcium sensors STIM1 and STIM2 control B cell regulatory function through interleukin‐10 production. Immunity 2011 34: 703–714. 21530328 10.1016/j.immuni.2011.03.016

[13] Mizoguchi, E. , Mizoguchi, A. , Preffer, F. I. and Bhan, A. K. , Regulatory role of mature B cells in a murine model of inflammatory bowel disease. Int. Immunol. 2000 12: 597–605. 10784605 10.1093/intimm/12.5.597

[14] Evans, J. G. , Chavez‐Rueda, K. A. , Eddaoudi, A. , Meyer‐Bahlburg, A. , Rawlings, D. J. , Ehrenstein, M. R. and Mauri, C. , Novel suppressive function of transitional 2 B cells in experimental arthritis. J. Immunol. 2007 178: 7868–7878. 17548625 10.4049/jimmunol.178.12.7868

[15] Mauri, C. , Gray, D. , Mushtaq, N. and Londei, M. , Prevention of arthritis by interleukin 10‐producing B cells. J. Exp. Med. 2003 197: 489–501. 12591906 10.1084/jem.20021293 PMC2193864

[16] Mauri, C. , Mars, L. T. and Londei, M. , Therapeutic activity of agonistic monoclonal antibodies against CD40 in a chronic autoimmune inflammatory process. Nat. Med. 2000 6: 673–679. 10835684 10.1038/76251

[17] Lampropoulou, V. , Hoehlig, K. , Roch, T. , Neves, P. , Calderon Gomez, E. , Sweenie, C. H. , Hao, Y. et al., TLR‐activated B cells suppress T cell‐mediated autoimmunity. J. Immunol. 2008 180: 4763–4773. 18354200 10.4049/jimmunol.180.7.4763

[18] Wang, K. , Tao, L. , Su, J. , Zhang, Y. , Zou, B. , Wang, Y. , Zou, M. et al., TLR4 supports the expansion of FasL(+)CD5(+)CD1d(hi) regulatory B cells, which decreases in contact hypersensitivity. Mol. Immunol. 2017 87: 188–199. 28505514 10.1016/j.molimm.2017.04.016

[19] Buenafe, A. C. and Bourdette, D. N. , Lipopolysaccharide pretreatment modulates the disease course in experimental autoimmune encephalomyelitis. J. Neuroimmunol. 2007 182: 32–40. 17055066 10.1016/j.jneuroim.2006.09.004

[20] Khan, A. R. , Amu, S. , Saunders, S. P. , Hams, E. , Blackshields, G. , Leonard, M. O. , Weaver, C. T. et al., Ligation of TLR7 on CD19(+) CD1d(hi) B cells suppresses allergic lung inflammation via regulatory T cells. Eur. J. Immunol. 2015 45: 1842–1854. 25763771 10.1002/eji.201445211

[21] Yoshizaki, A. , Miyagaki, T. , DiLillo, D. J. , Matsushita, T. , Horikawa, M. , Kountikov, E. I. , Spolski, R. et al., Regulatory B cells control T‐cell autoimmunity through IL‐21‐dependent cognate interactions. Nature 2012 491: 264–268. 23064231 10.1038/nature11501 PMC3493692

[22] Wang, R. X. , Yu, C. R. , Dambuza, I. M. , Mahdi, R. M. , Dolinska, M. B. , Sergeev, Y. V. , Wingfield, P. T. et al., Interleukin‐35 induces regulatory B cells that suppress autoimmune disease. Nat. Med. 2014 20: 633–641. 24743305 10.1038/nm.3554 PMC4048323

[23] Shen, H. , Wang, C. , Fan, E. , Li, Y. , Zhang, W. and Zhang, L. , Upregulation of interleukin‐35 subunits in regulatory T cells in a murine model of allergic rhinitis. ORL J. Otorhinolaryngol. Relat. Spec. 2014 76: 237–247. 25412964 10.1159/000369141

[24] Walters, S. , Webster, K. E. , Sutherland, A. , Gardam, S. , Groom, J. , Liuwantara, D. , Marino, E. et al., Increased CD4+Foxp3+ T cells in BAFF‐transgenic mice suppress T cell effector responses. J. Immunol. 2009 182: 793–801. 19124722 10.4049/jimmunol.182.2.793

[25] Yang, M. , Sun, L. , Wang, S. , Ko, K. H. , Xu, H. , Zheng, B. J. , Cao, X. et al., Novel function of B cell‐activating factor in the induction of IL‐10‐producing regulatory B cells. J. Immunol. 2010 184: 3321–3325. 20208006 10.4049/jimmunol.0902551

[26] Hua, C. , Audo, R. , Yeremenko, N. , Baeten, D. , Hahne, M. , Combe, B. , Morel, J. et al., A proliferation inducing ligand (APRIL) promotes IL‐10 production and regulatory functions of human B cells. J. Autoimmun. 2016 73: 64–72. 27372914 10.1016/j.jaut.2016.06.002

[27] Matsumoto, M. , Baba, A. , Yokota, T. , Nishikawa, H. , Ohkawa, Y. , Kayama, H. , Kallies, A. et al., Interleukin‐10‐producing plasmablasts exert regulatory function in autoimmune inflammation. Immunity 2014 41: 1040–1051. 25484301 10.1016/j.immuni.2014.10.016

[28] McNab, F. , Mayer‐Barber, K. , Sher, A. , Wack, A. and O'Garra, A. , Type I interferons in infectious disease. Nat. Rev. Immunol. 2015 15: 87–103. 25614319 10.1038/nri3787 PMC7162685

[29] Gonzalez‐Navajas, J. M. , Lee, J. , David, M. and Raz, E. , Immunomodulatory functions of type I interferons. Nat. Rev. Immunol. 2012 12: 125–135. 22222875 10.1038/nri3133 PMC3727154

[30] Ivashkiv, L. B. and Donlin, L. T. , Regulation of type I interferon responses. Nat. Rev. Immunol. 2014 14: 36–49. 24362405 10.1038/nri3581 PMC4084561

[31] McFarlane, A. J. , McSorley, H. J. , Davidson, D. J. , Fitch, P. M. , Errington, C. , Mackenzie, K. J. , Gollwitzer, E. S. et al., Enteric helminth‐induced type I interferon signaling protects against pulmonary virus infection through interaction with the microbiota. J. Allergy Clin. Immunol. 2017 140: 1068–1078.e6. 28196762 10.1016/j.jaci.2017.01.016 PMC6485385

[32] Trottein, F. , Pavelka, N. , Vizzardelli, C. , Angeli, V. , Zouain, C. S. , Pelizzola, M. , Capozzoli, M. et al., A type I IFN‐dependent pathway induced by Schistosoma mansoni eggs in mouse myeloid dendritic cells generates an inflammatory signature. J. Immunol. 2004 172: 3011–3017. 14978105 10.4049/jimmunol.172.5.3011

[33] Webb, L. M. , Lundie, R. J. , Borger, J. G. , Brown, S. L. , Connor, L. M. , Cartwright, A. N. , Dougall, A. M. et al., Type I interferon is required for T helper (Th) 2 induction by dendritic cells. EMBO J. 2017 36: 2404–2418. 28716804 10.15252/embj.201695345 PMC5556270

[34] Connor, L. M. , Tang, S. C. , Cognard, E. , Ochiai, S. , Hilligan, K. L. , Old, S. I. , Pellefigues, C. et al., Th2 responses are primed by skin dendritic cells with distinct transcriptional profiles. J. Exp. Med. 2017 214: 125–142. 27913566 10.1084/jem.20160470 PMC5206495

[35] Braun, D. , Caramalho, I. and Demengeot, J. , IFN‐alpha/beta enhances BCR‐dependent B cell responses. Int. Immunol. 2002 14: 411–419. 11934877 10.1093/intimm/14.4.411

[36] Poovassery, J. S. and Bishop, G. A. , Type I IFN receptor and the B cell antigen receptor regulate TLR7 responses via distinct molecular mechanisms. J. Immunol. 2012 189: 1757–1764. 22786773 10.4049/jimmunol.1200624

[37] Kiefer, K. , Oropallo, M. A. , Cancro, M. P. and Marshak‐Rothstein, A. , Role of type I interferons in the activation of autoreactive B cells. Immunol. Cell Biol. 2012 90: 498–504. 22430248 10.1038/icb.2012.10 PMC3701256

[38] Crow, M. K. , Type I interferon in the pathogenesis of lupus. J. Immunol. 2014 192: 5459–5468. 24907379 10.4049/jimmunol.1002795 PMC4083591

[39] Menon, M. , Blair, P. A. , Isenberg, D. A. and Mauri, C. , A regulatory feedback between plasmacytoid dendritic cells and regulatory B cells is aberrant in systemic lupus erythematosus. Immunity 2016 44: 683–697. 26968426 10.1016/j.immuni.2016.02.012 PMC4803914

[40] Kozovska, M. E. , Hong, J. , Zang, Y. C. , Li, S. , Rivera, V. M. , Killian, J. M. and Zhang, J. Z. , Interferon beta induces T‐helper 2 immune deviation in MS. Neurology 1999 53: 1692–1697. 10563614 10.1212/wnl.53.8.1692

[41] Liu, H. , MacKenzie‐Graham, A. J. , Kim, S. and Voskuhl, R. R. , Mice resistant to experimental autoimmune encephalomyelitis have increased thymic expression of myelin basic protein and increased MBP specific T cell tolerance. J. Neuroimmunol. 2001 115: 118–126. 11282161 10.1016/s0165-5728(01)00269-7

[42] Schubert, R. D. , Hu, Y. , Kumar, G. , Szeto, S. , Abraham, P. , Winderl, J. , Guthridge, J. M. et al., IFN‐beta treatment requires B cells for efficacy in neuroautoimmunity. J. Immunol. 2015 194: 2110–2116. 25646307 10.4049/jimmunol.1402029 PMC4340715

[43] Mangan, N. E. , van Rooijen, N. , McKenzie, A. N. and Fallon, P. G. , Helminth‐modified pulmonary immune response protects mice from allergen‐induced airway hyperresponsiveness. J. Immunol. 2006 176: 138–147. 16365404 10.4049/jimmunol.176.1.138

[44] Smits, H. H. , Hammad, H. , van Nimwegen, M. , Soullie, T. , Willart, M. A. , Lievers, E. , Kadouch, J. et al., Protective effect of Schistosoma mansoni infection on allergic airway inflammation depends on the intensity and chronicity of infection. J. Allergy Clin. Immunol. 2007 120: 932–940. 17689595 10.1016/j.jaci.2007.06.009

[45] Fitzgerald‐Bocarsly, P. , Dai, J. and Singh, S. , Plasmacytoid dendritic cells and type I IFN: 50 years of convergent history. Cytokine Growth Factor Rev. 2008 19: 3–19. 18248767 10.1016/j.cytogfr.2007.10.006 PMC2277216

[46] van der Vlugt, L. E. P. M. , Labuda, L. A. , Ozir‐Fazalalikhan, A. , Lievers, E. , Gloudemans, A. K. , Liu, K.‐Y. , Barr, T. A. et al., Schistosomes induce regulatory features in human and mouse CD1dhi B cells: inhibition of allergic inflammation by IL‐10 and regulatory T cells. PLoS One 2012 7: e30883. 22347409 10.1371/journal.pone.0030883 PMC3275567

[47] Everts, B. , Perona‐Wright, G. , Smits, H. H. , Hokke, C. H. , van der Ham, A. J. , Fitzsimmons, C. M. , Doenhoff, M. J. et al., Omega‐1, a glycoprotein secreted by Schistosoma mansoni eggs, drives Th2 responses. J. Exp. Med. 2009 206: 1673–1680. 19635864 10.1084/jem.20082460 PMC2722183

