# Supplementary material for: Type I interferons provide additive signals for murine regulatory B cell induction by Schistosoma mansoni eggs
Source: Eur J Immunol. 2019 May 29;49(8):1226–34. doi: 10.1002/eji.201847858 (PMC6771625; doi:10.1002/eji.201847858)
Supplement: Supplementary file 1 — Supplemental Information [file EJI-49-1226-s001.pdf]

European Journal of Immunology

Supporting Information

for

DOI 10.1002/eji.201847858

Katja Obieglo, Alice Costain, Lauren M. Webb, Arifa Ozir-Fazalalikhan,
Shelia L. Brown, Andrew S. MacDonald and Hermelijn H. Smits

Type I interferons provide additive signals for murine regulatory B cell induction by *Schistosoma mansoni* eggs

© 2019 The Authors. *European Journal of Immunology* published by WILEY-VCH Verlag GmbH & Co. KGaA, Weinheim.

Supplemental Information

Type I interferons provide additive signals for murine regulatory B cell induction by *Schistosoma* mansoni eggs

Katja Obieglo, Alice Costain, Lauren M. Webb, Arifa Ozir-Fazalalikhan, Shelia L. Brown, Andrew S. MacDonald, Hermelijn H. Smits

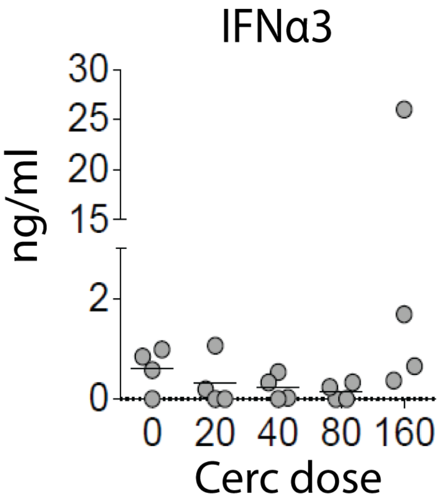

Suppl. Figure 1:

A trend towards enhanced IFNα3 serum levels in mice infected with 160 cercariae.

Mice were infected with 20-160 cercariae and serum samples were taken at d56 of infection for assessment of cytokines by ELISA. Data from 1 experiment, with 4 mice per group.

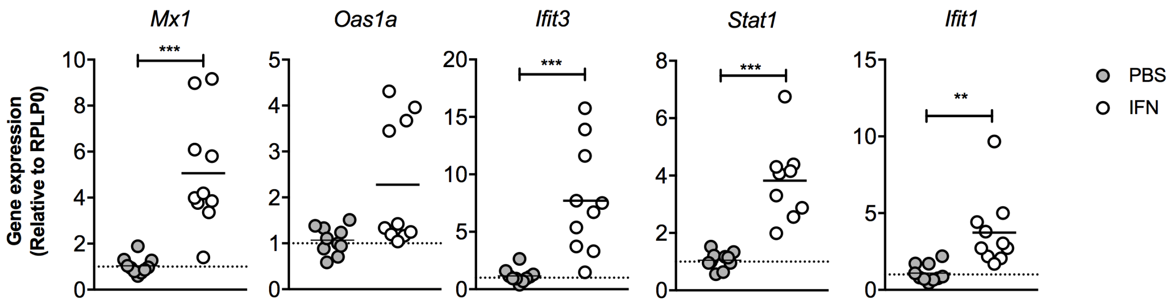

Suppl. Figure 2: Expression of interferon stimulated genes in IFNα injected mice 12 hours post-injection. Splenocytes were isolated from PBS- and IFNα-treated mice**.** The mRNA expression of interferon-stimulated genes (ISGs) was assessed by qPCR (relative to RPLP0). N=8 per group. Significant differences were determined by unpaired t-test. * $p < 0.05$, ** $p < 0.01$, *** $p < 0.001$.

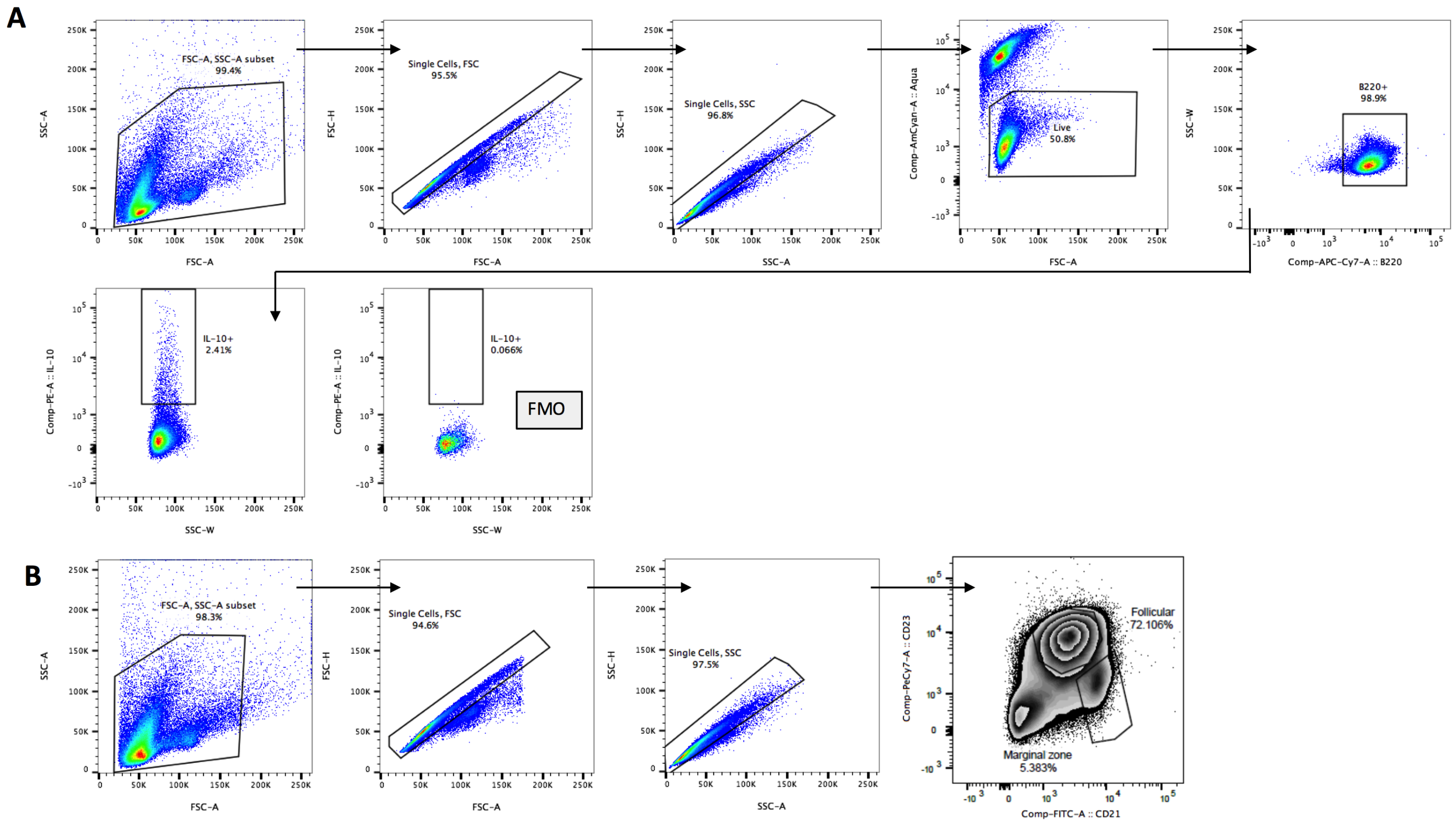

Suppl. Figure 3. Flow cytometry gating schemes. (A) Identification of IL-10 production from 3-day cultured B cells. **(B)** Cell sorting scheme for follicular and marginal zone B cell subsets from MACs Isolated B cell.

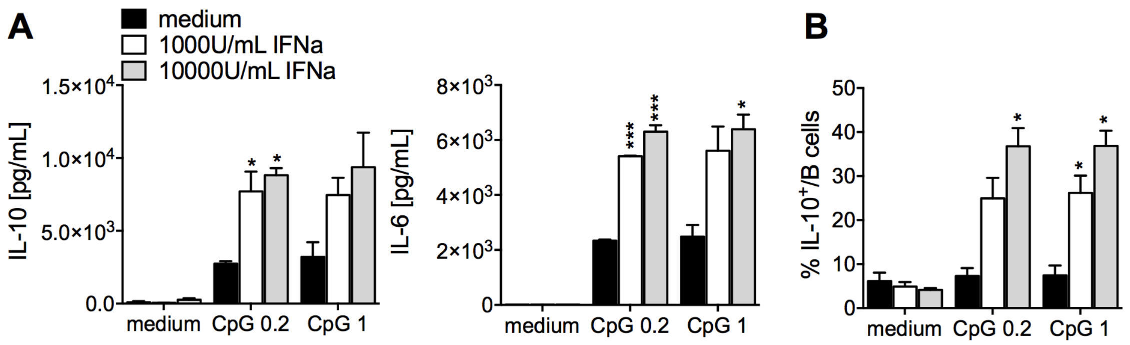

Suppl. Figure 4: Recombinant IFNα enhances CpG-induced B cell IL-10 and IL-6 production. B cells were isolated from the spleen of naïve mice and stimulated *in vitro* with CpG ODN1826 (class B; 0.2-1μM) and IFNα (10^3-10^4 U/mL) as indicated. After 3 days of culture, supernatants were analyzed for IL-10 and IL-6 concentration by ELISA (**A**), and % IL-10 B cells assessed by flow cytometry (**B**). Summary of 2-3 experiments, each data point is the mean of two technical replicates. Data are presented as mean ± SEM. Significant differences were determined by one-way ANOVA followed by Dunnett's multiple comparisons test. * $p < 0.05$, *** $p < 0.001$.

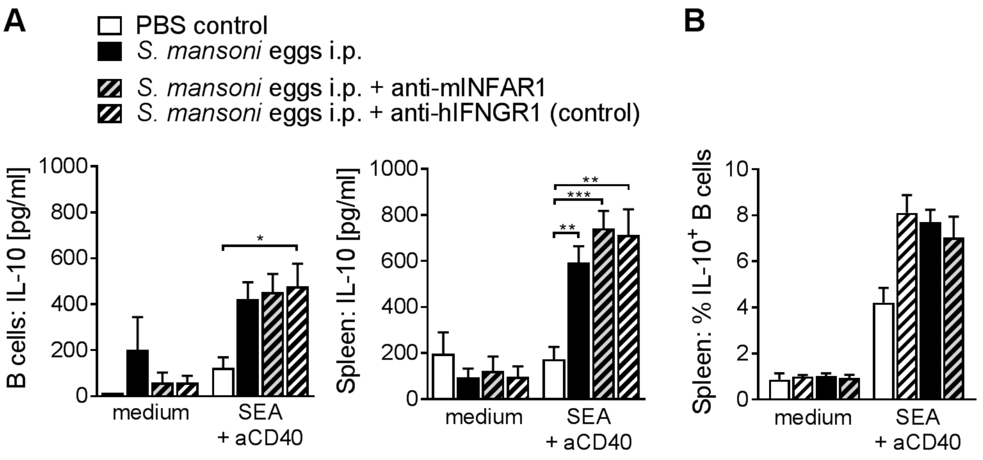

Suppl. Figure 5: IFNAR1 signaling is dispensable for Breg cell induction *in vivo*. Mice were treated as depicted in **A.** On day 14, spleens were harvested and total splenocyte cell suspensions and isolated $CD19^+$ B cells restimulated with SEA (20ug/ml) and anti-CD40 (2ug/ml) for 2 days. Supernatants were analyzed for IL-10 and IL-6 concentration by ELISA (**B**), and the percentage of IL-10^+ B cells assessed by flow cytometry (**C**). Data from one experiment, n=5/group. Data are presented as mean ± SEM. Significant differences were determined by one-way ANOVA followed by Tukey's multiple comparisons test. * $p < 0.05$, ** $p < 0.01$, *** $p < 0.001$.
